# The clinical validity of radiomics-based prediction of molecular subtypes in breast cancer from digital mammary tomosynthesis

**DOI:** 10.3389/fonc.2025.1661116

**Published:** 2025-10-13

**Authors:** Jing Xue, Yilun Li, Tianyun Qu, Yidi Qin, Haoqi Wang, Xiaocui Rong, Jingliu Tian, Tao Wang, Jianhua Zhang, Zhigang Li, Yong Ping

**Affiliations:** ^1^ Department of Radiology, The Fourth Hospital of Hebei Medical University, Shijiazhuang, Hebei, China; ^2^ Department of Breast surgery, The Fourth Hospital of Hebei Medical University, Shijiazhuang, Hebei, China; ^3^ Department of Radiology, Shijiazhuang Hospital of Traditional Chinese Medicine, Shijiazhuang, Hebei, China; ^4^ Department of Imaging Department, Baixiang County Central Hospital, Xingtai, Hebei, China; ^5^ Department of Radiology, Xiongxian Hospital, Baoding, Hebei, China

**Keywords:** breast cancer, imagomics, diagnosis, nomogram model, molecular subtypes

## Abstract

**Objective:**

To explore the use of digital breast tomography (DBT) imaging omics in developing breast cancer (BC) diagnostic models to identify molecular subtype characteristics of BC.

**Methods:**

A retrospective analysis was conducted on 433 DBT images. Candidate features were extracted, and least absolute shrinkage and selection operator (LASSO) regression model was established. Within the training set, machine learning (ML) models were constructed, and their predictive performance was evaluated using receiver operating characteristic (ROC) curves and confusion matrixes in the test set, thereby screening the best predictive classifier. Univariate and multivariate Cox regression analyses were conducted to obtain key characteristics of nomogram modeling, correction and decision curve analysis (DCA) were used to evaluate the clinical potential of this model.

**Results:**

The LASSO selected 14 features. Random Forest (RF) had the highest AUC value, the highest accuracy, sensitivity, recall rate and F1 score on the training set and test set, and was the best classifier. A nomogram model was established. The odds ratio (OR) of BC patients increased with the increase of the total score.

**Conclusion:**

The key features of BC were revealed by image omics and ML models, and a nomogram model with diagnostic value was constructed.

## 1 Introduction

Breast cancer (BC) is the most common cancer diagnosed in women, and the second leading cause of all cancer-related deaths (1). Early diagnosis of BC, as well as predicting prognosis and treatment response, is a primary focus of clinical research. Based on specific receptor expression levels, BC subtypes include luminal, human epidermal growth factor receptor 2 (HER2)-enriched, and triple-negative (TN) (2, 3). TN BC is notably more aggressive and untreatable with endocrine therapy or trastuzumab, while its distinct MRI patterns can be quantified via radiomics for precise subtype diagnosis (2, 4–6).

Unlike genomic/transcriptomic profiling, which often analyzes limited tumor samples, radiomics assesses whole-tumor heterogeneity (7–9). While mammography, ultrasonography, and MRI findings correlate with molecular subtypes (10–12), recent efforts focus on predicting them radiomically, which extracts high-dimensional, quantitative features from images, capturing both tissue characteristics and gene expression profiles (13). Digital breast tomography (DBT) has become the breast imaging standard: adding DBT to digital mammography increases cancer detection rates versus mammography alone (14, 15). Although MRI excels in tissue characterization, its routine use remains limited (16, 17). Thus, non-invasive subtype prediction using widely available DBT has significant clinical value—it avoids invasive biopsies that cause patient discomfort, reduces the risk of complications from invasive procedures, and enables early, precise subtype-guided treatment.

Despite DBT’s role in BC diagnosis, challenges like increased reading workload and inconsistent mass segmentation (due to numerous image slices) limit radiomic application (18). Synthetic mammography offers a solution for integrating radiomics into clinical practice. However, prior radiomic studies focused on MRI (costly/non-routine) or conventional mammography, with scarce research on DBT-radiomics for subtype prediction, and radiomics-ML integration remains underdeveloped.

Some studies have explored the use of radiomic methods for analyzing molecular subtypes in DBT-derived synthetic mammography. In the study by Xiong et al., which focused on patients with invasive BC, radiomic features proved effective in predicting disease-free survival (DFS) and outperformed clinicopathological nomograms (19). Some investigations have shown that radiomic features derived from magnetic resonance imaging (MRI) correlate with the molecular subtypes of BC (20–22). While MRI combined with radiomics has greatly contributed to personalized BC treatment, there is currently a lack of research on using DBT imaging radiomics to predict the molecular subtypes of BC. However, the research on imaging omics and machine learning to build diagnostic models to predict diseases in BC is not deep enough, and it is worth further exploration.

In our study, a variety of machine learning algorithms were applied to establish a combined model of DBT imaging omics and immunohistochemistry (IHC) data, and 5 high-risk characteristics of BC were screened, which provided more valuable information to the clinic and were conducive to personalized clinical treatment.

## 2 Materials and methods

### 2.1 Patients

**Figure 1:**
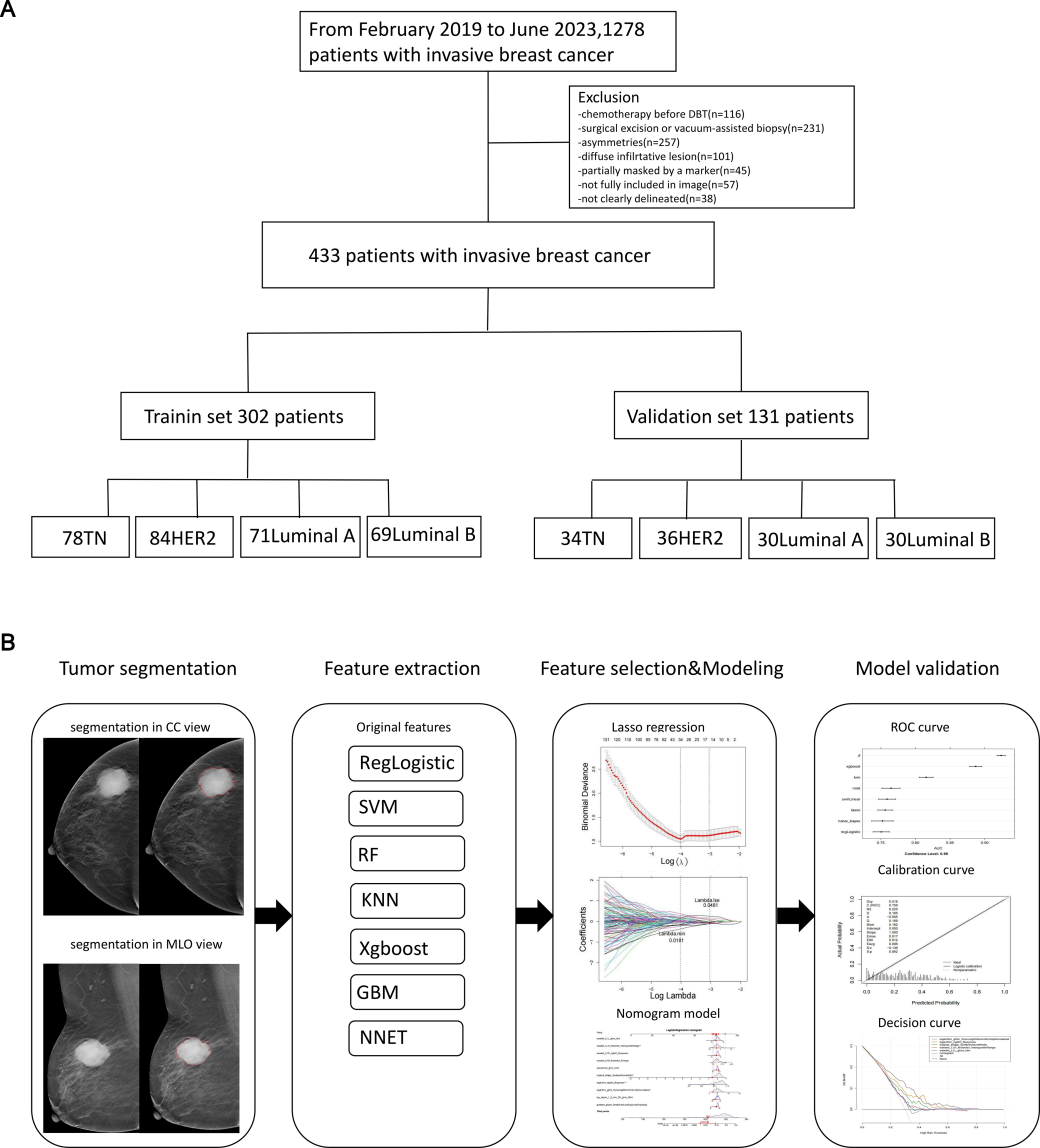
The flowchart of the study. This flowchart outlines the key steps of the study. **(A)** Patient selection process. **(B)** Workflow for constructing and validating the radiomics nomogram.

This retrospective study, approved by our Institutional Review Board (IRB) with waived written consent, identified 433 consecutive female patients who were diagnosed with invasive BC and had available preoperative mammography at our institution between February 2019 and June 2023. This study has been approved by the Ethics Committee of S&T Program of Hebei (20377783D), Medical Science Research Project of Hebei (20221342), and Medical Science Research Project of Hebei (20230863)). All patients included in the study met the following inclusion criteria: (1) underwent DBT examination within one month prior to surgery; (2) were pathologically diagnosed with invasive breast carcinoma; (3) had no documented history of any other malignancy; and (4) did not undergo a biopsy or receive treatment for the breast tumor before the DBT examination. Patients were excluded from the study if they met any of the following exclusion criteria: (1) insufficient clinicopathological data or suboptimal image quality; (2) pathological diagnosis of non-invasive breast carcinoma, or concurrent presence of other malignancies; (3) multiple BC lesions or distant metastases; (4) tumors on DBT images that did not appear as masses but presented in other forms, such as pure calcification, asymmetry, or architectural distortion. The 433 cases included 111 patients with subtype A BC, 100 patients with subtype B BC, 107 patients with HER2-positive BC, and 112 patients with basal-like BC. These cases were used to screen for regions of interest (ROI) and extract image-omics features (**Figure 1A**).

### 2.2 DBT examination

In this study, the patients were scanned using GE Senographe Essential digital mammography (GE Healthineers) fullfield digital mammography systems. The typical imaging parameters were established within the ranges of 27–32 kV and 28–68 mAs. Additionally, both craniocaudal and mediolateral oblique images were successfully obtained for every patient. This imaging technique facilitated the acquisition of both a standard digital mammogram and a tomosynthesis scan under the same breast compression (23, 24). With a single low-dose exposure, the X-ray tube was rotated through an angular range of 12.5 degrees, completing a total of nine rotations. Advanced computerized imaging algorithms were then employed to reconstruct projections from each viewing angle, enabling three-dimensional visualization of breast tissue.

### 2.3 Image information

**Figure 2:**
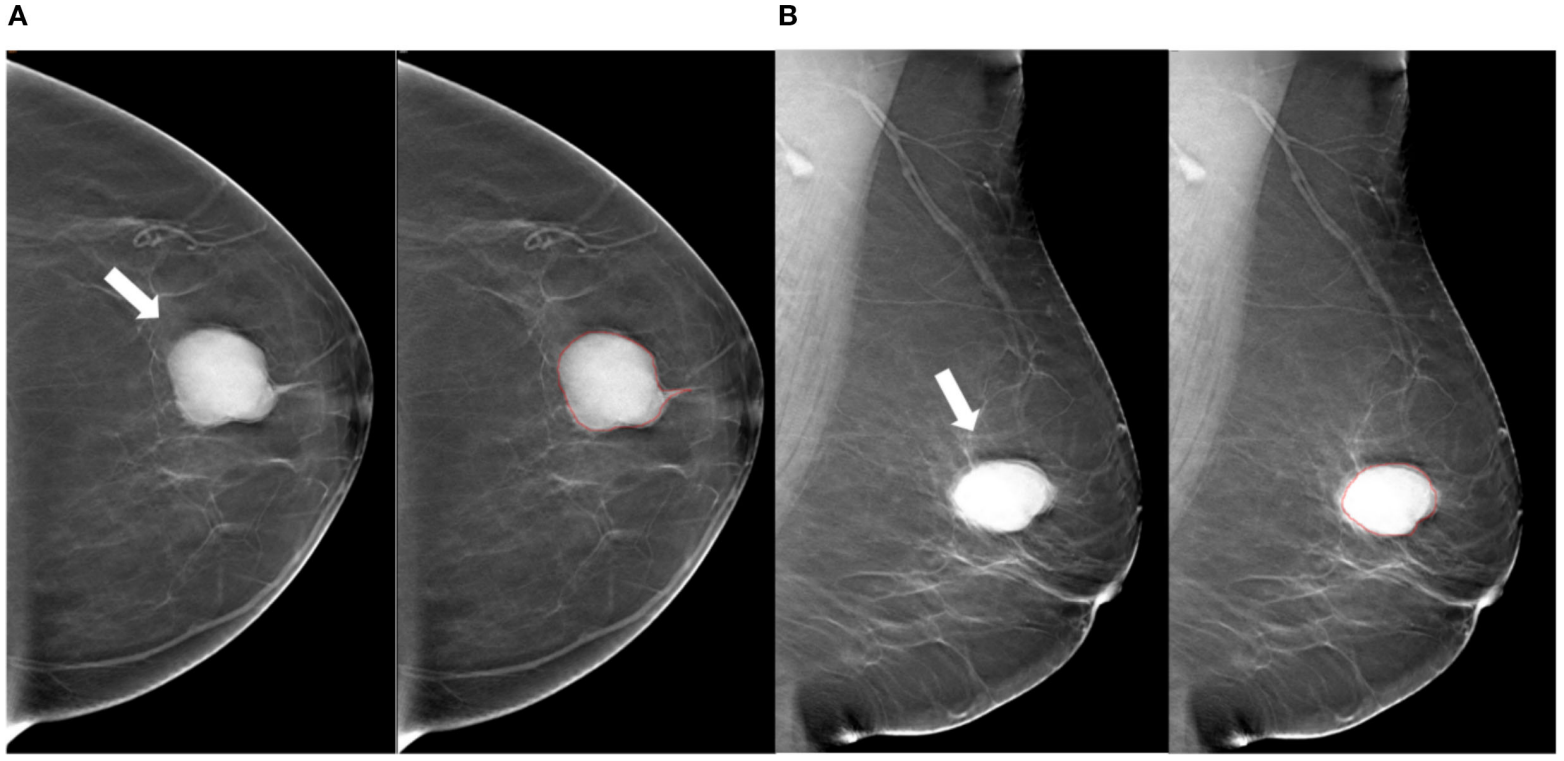
Tumor segmentation example 1. Example of tumor segmentation on synthetic mammography. The synthetic mediolateral oblique **(A)** and craniocaudal **(B)** views of a 69-year-old female diagnosed with the luminal A subtype of breast cancer. The breast lesion appears as a circumscribed and round mass with high density (arrow).

**Figure 3:**
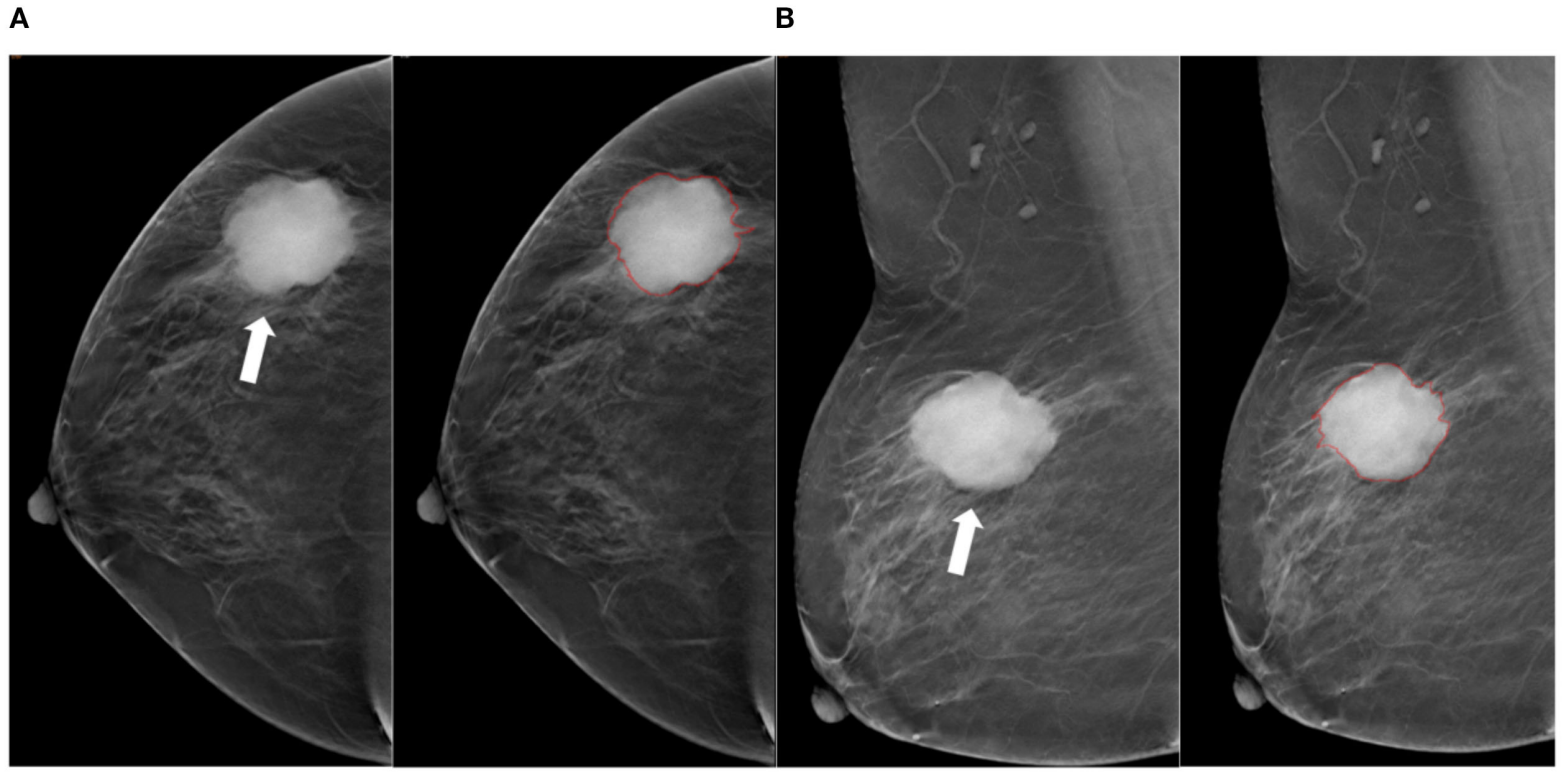
Tumor segmentation example 2. Example of tumor segmentation on synthetic mammography. The synthetic mediolateral oblique **(A)** and craniocaudal **(B)** views of a 79-year-old female diagnosed with the triple-negative subtype of breast cancer. The breast lesion appears as a spiculated mass (arrow).

Two experienced radiologists, each with more than 5 years of professional expertise, performed an impartial evaluation of DBT images, which were anonymized. The three-dimensional ROI that encompassed the tumor on synthetic mammography was manually segmented (as shown in **Figures 2**, **3**) by a resident radiologist with five years of experience (referred to as reader 1) using the “3D Slicer” software (https://www.slicer.org/). Subsequently, the delineated ROIs were meticulously examined and verified by a breast radiologist who possessed a decade of subspecialty experience (referred to as reader 2). In cases where there were discrepancies regarding the ROI, they were resolved through consensus-based discussions. This assessment was conducted in a blinded manner, meaning that the radiologists were not provided access to the associated histopathological information to ensure an unbiased judgment. In instances where discrepancies arose between the two radiologists, a third radiologist with more than 10 years of experience was consulted to resolve the discrepancies.

### 2.4 Pathological and IHC analysis

Surgical resection of BC specimens was performed, followed by validation of diagnoses through histopathological examination. IHC analyses were conducted to assess the expression levels of estrogen receptor (ER), progesterone receptor (PR), human epidermal growth factor receptor 2 (HER-2), and the Ki-67 antigen (25, 26). The absence of positive staining in 1% or fewer carcinoma nuclei indicated a negative status for both ER and PR, as per references (26, 27). According to the IHC scoring system, HER-2 expression was categorized into four levels: 0, 1+, 2+, or 3 +. Confirmation of a negative HER-2 status can be achieved through two methods: obtaining an IHC score of either 0 or 1+, or achieving an IHC score of 2+ alongside a negative result from fluorescence *in situ* hybridization (FISH) testing. Conversely, a positive HER-2 status can be confirmed with either a score of 3+ or a score of 2+ combined with a positive FISH test result (27, 28). Furthermore, if a patient presented with an IHC score of 2+ but lacked FISH results, the recorded status for HER-2 was classified as suspicious positive. A Ki-67 proliferation index below 14% was categorized as a low level of proliferation, while a value equal to or exceeding 14% was regarded as a high level of proliferation (28). The IHC antibodies used in this study were as follows: ER (Roche, Clone Number SP1), PR (Roche, Clone number IE2), HER2 (Roche, Clone number 4B5), and KI-67 (Maxin, Clone number MX006).

### 2.5 Lesion segmentation and feature extraction

The ROI on the cranio-caudal (CC) and mediolateral oblique (MLO) views of DBT images was manually delineated, following the contours of the tumor’s maximum diameter area. The lesion segmentation task was conducted by two radiologists, referred to as Radiologist 1 and Radiologist 2, who possessed 10 and 7 years of experience in BC diagnosis, respectively. They employed 3D Slicer (version 4.11; available at http://www.slicer.org) to outline all ROIs while remaining unaware of the histopathological data during this process. **Figure 1B** presents a diagrammatic representation illustrating the segmentation of the ROI. Prior to commencing feature extraction, the images underwent resampling and grayscale discretization for normalization purposes, adhering to recommendations established in prior research studies (28, 29). Radiomic features were extracted from both CC and MLO images encompassing each patient’s ROI.

### 2.6 Selecting of radiomics features and establishment of the radscore model

Radiomic feature extraction was performed using Python (version 3.7) of the PyRadiomics package (version 3.0.1, http://pyradiomics.readthedocs.io), according to the original, wavelet, gauss Laplace (LoG), index, square, square root, logarithm and gradient image retrieval. These image omics features were statistically processed by Z-score and Kruskal-Wallis rank sum test to screen out candidate features (*p* < 0.05).

For the sake of screening features for predicting BC, the dataset was randomly split into training and validation subsets at a 7:3 ratio. The radiomics features were normalized via Z-score standardization (28). To precisely identify the most effective set of predictive features, the study utilized least absolute shrinkage and selection operator (LASSO) regression, implementing five-fold cross-validation. The One-vs-Rest (OvR) strategy was utilized for predicting the sample subtypes via building a binary classifier. Based on the training set, the LASSO regression model was then constructed by the R package ‘glmnet’ (Ver. 4.1-6) to select LASSO features with the parameters of ‘famil’=‘binomial’ and ‘type.measure’=‘class’. The results of multivariate classification were determined by taking the category with the highest probability (HER2-positive) through 10-fold cross-validation. And the LASSO features were ascertained when the error rate of model was lowest. Moreover, the receiver operating characteristic (ROC) curves were plotted in training and testing set to evaluate the performance of the model in predicting the subtypes of BC (area under of ROC curves (AUCs) > 0.70).

In order to assess the correlations between LASSO features and BC subtypes, Dunn’s test was exploited to determine whether there were significant discrepancies in LASSO features between luminal A-subtype, luminal B-subtype, HER2-positive BC and basal-like BC. The violin plots were created via the R package ‘ggstatsplot’ (Ver. 0.12.0) to exhibit the outcomes.

### 2.7 Selecting of the optimal classifier and nomogram modeling

To screen the optimal classifier for further predicting BC subtypes using image-omics LASSO features, 8 machine learning (ML) models in the R package ‘caret’ (Ver. 6.0-94) were constructed. In the training set, Regularized Logistic Regression (regLogistic), support vector machine (SVM), Random Forest (RF), k-nearest neighbors (KNN), eXtreme Gradient Boosting (xgboost), Gradient Boosting Machine (GBM), Naive Bayes and Neural Network (NNET) models were constructed to predict the categories of samples, and the diagnostic efficacy of each model was calculated separately with the 5-fold cross-validation. The predictive performances of the 8 models were evaluated with ROC curve and confusion matrix in training and testing sets, and AUCs of 8 models in the training and testing sets were compared. Simultaneously, accuracy, sensitivity, specificity, recall, precision and F1 score of each model were estimated to filter the classifier with the best performance. Eventually, the optimal classifier was used to rank the importance of each LASSO feature. Ulteriorly, univariate (*p* < 0.05) and multivariate Cox regression analyses (*p* < 0.05) were proceeded through the R package ‘rms’ (Ver. 6.5-0) to acquire crucial features for nomogram modeling. The relationships between crucial features and BC were predicted in the light of the odds ratio (OR) of the patients in the nomogram model. Additionally, calibration curve and Decision Curve Analysis (DCA) were adopted to evaluate the predictive power of nomogram model.

### 2.8 Statistical analysis

Bioinformatics analyses were conducted using R software (Version 6.0-94). Significant differences among three or more groups were assessed by the Kruskal-Wallis rank sum test, followed by Dunn’s test for pairwise comparisons between multiple groups. A *p*-value or adjusted *p*-value (p.adj) of less than 0.05 was considered statistically significant.

## 3 Results

### 3.1 A sum of 14 LASSO features were obtained

**Figure 4:**
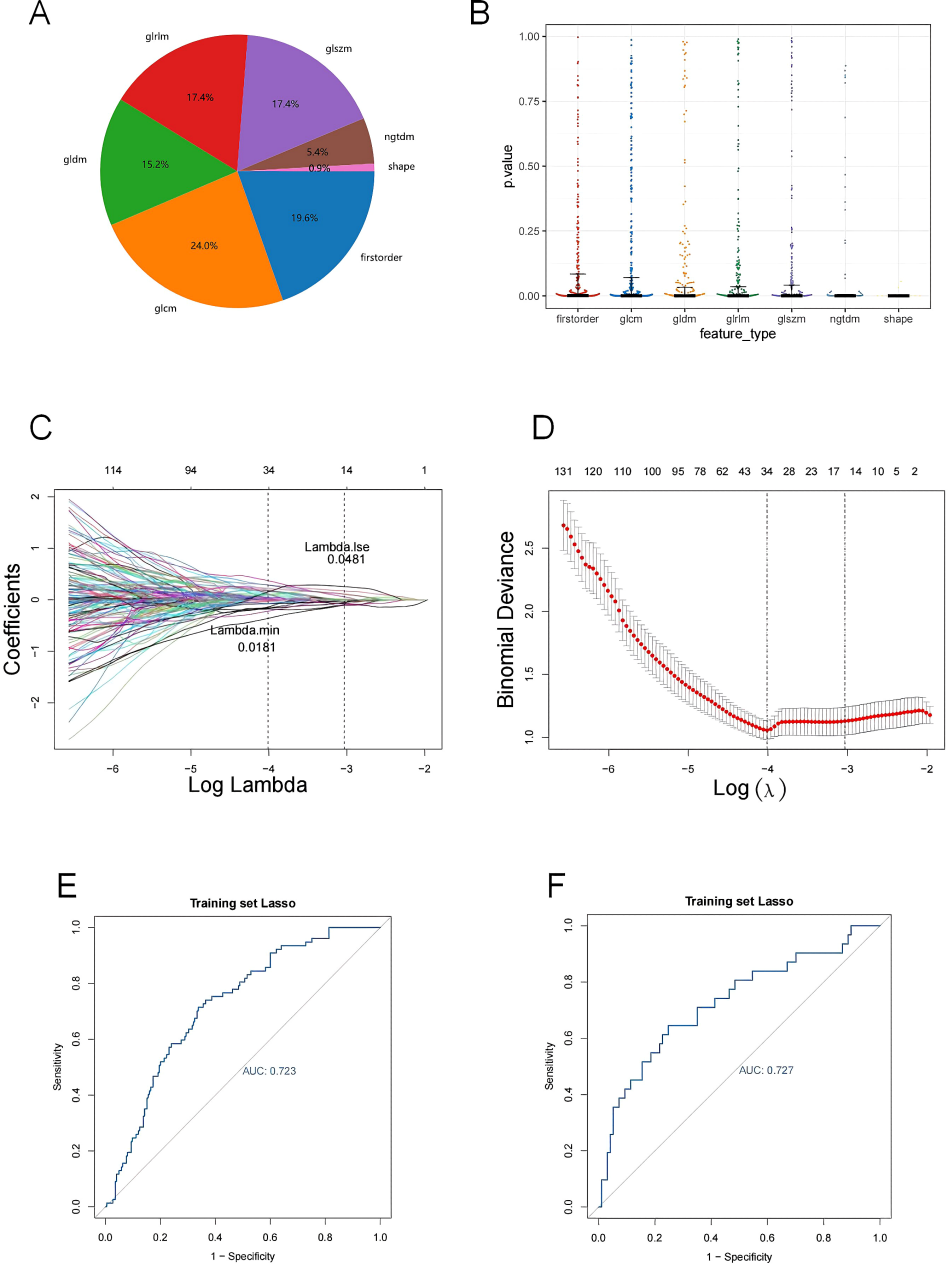
**(A)** The proportion of extracted features; **(B)** P-value distribution of statistical tests of imaging omics features; **(C, D)** Feature screening by LASSO logistic regression; **(E, F)** Model ROC curves for validation and test sets. **(A)** The percentage of each feature extracted by LASSO model; **(B)** Kruskal-Wallis rank sum test was performed on all image omics features, and only the image omics features with a p value less than 0.05 were retained; **(C)** Characteristic coefficient variations with penalty coefficient; **(D)** Cross-verify error plots. Figure (left): The horizontal coordinate is Lambda, and the vertical coordinate represents the error of cross-validation. In the actual analysis, we expect the position with the smallest mean square error of cross-validation, and the left dotted line is the position with the smallest cross-validation error. According to the position lambda.min, the corresponding horizontal coordinate lambda is determined and the optimal Lambda value is found. The minimum Lambda value of 0.0481 produces 14 noose features. The size of the mean square error of the model is shown in the figure on the right; **(E)** Verify the model ROC curve of the set; **(F)** Test the model ROC curve of the set. The model ROC curve AUC of the verification set and the test set is greater than 0.7.

**Figure 5:**
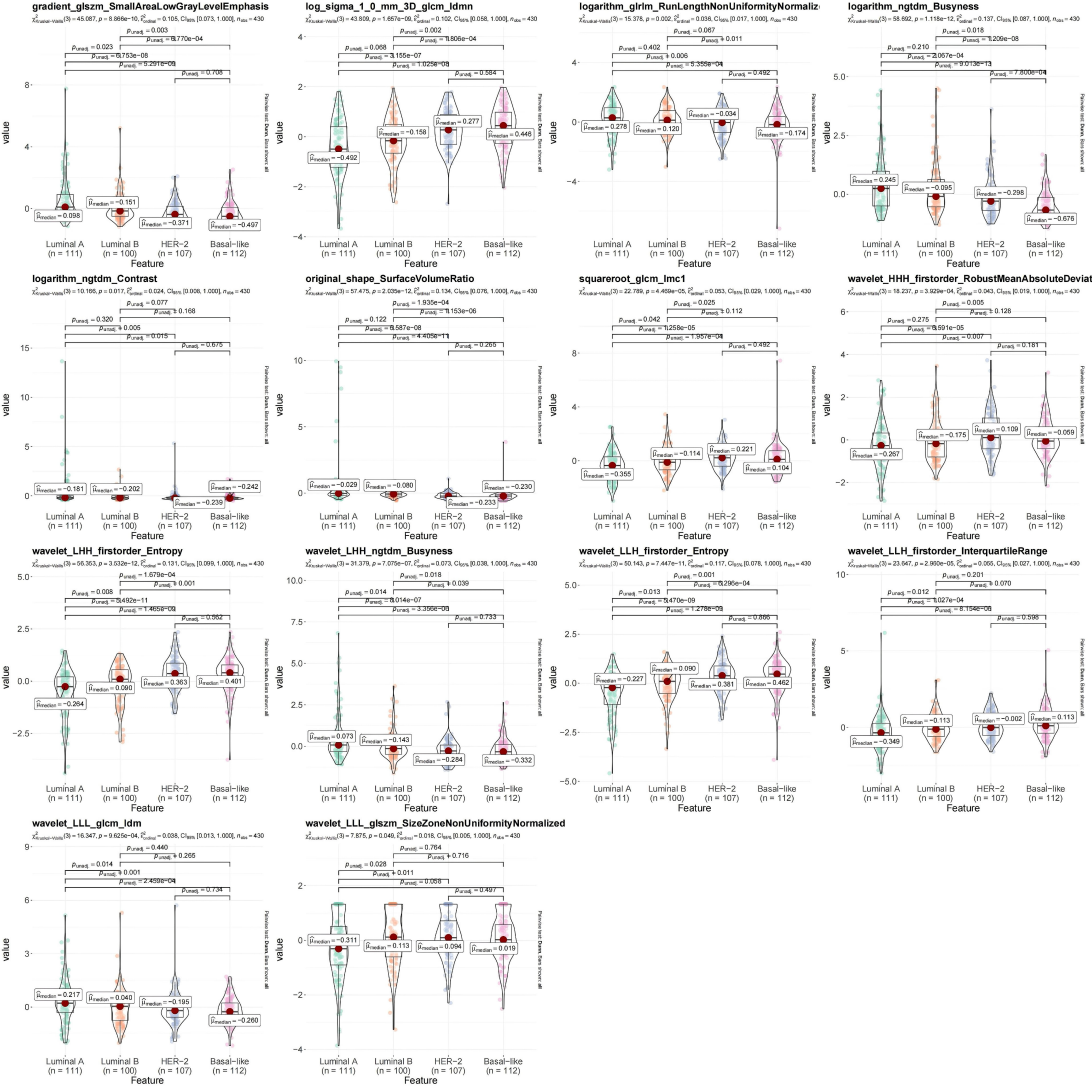
Violin diagram of strong correlation between different types of breast cancer (BC). The R package ggstatsplot (version 0.12.0) was used to plot the strong correlation features between different BC subtypes, evaluate the correlation between the strong correlation features and BC subtypes, and show the most common *post-hoc* test after the important Kruskal-Wallis test - Dunn test. P<0.05 indicates that there are significant differences in the strong correlation characteristics between different clinical groups.

A total of 7 types of features, encompassing 306 first-order features, 14 shape features, and 1,241 texture features (glcm, gldm, glrlm, glszm and ngtdm) emerged from the original, wavelet, LoG, exponential, square, squareRoot, logarithm, gradient images (**Table 1**, **Figure 4A**). In sum of 1,175 candidate features were obtained after Z-score standardization and Kruskal-Wallis rank sum test for 7 types (**Figure 4B**). In order to screen features that were strongly associated with BC, a LASSO regression model was built in the training set, producing 14 LASSO features when the minimum Lambda value was 0.0481 (**Figures 4C, D**). The AUCs of the training and testing sets were 0.723 and 0.727, respectively (**Figures 4E, F**). The correlation analysis between LASSO features and BC subtypes revealed significant differences among the 14 LASSO features across different subtype comparisons. Specifically, between luminal B and HER2-positive subtypes, the following nine features showed significant differences: gradient glszm SmallAreaLowGrayLevelEmphasis, log sigma 1–0 mm 3D glcm Idmn, logarithm ngtdm Busyness, squareroot glcm Imc1, wavelet HHH firstorder RobustMeanAbsoluteDeviat, wavelet LHH ngtdm Busyness, wavelet LLH firstorder Entropy, wavelet LLL glcm Idm and wavelet LLL glszm SizeZoneNonUniformityNormalized. Additionally, between luminal A and luminal B subtypes, eight features exhibited significant differences: gradient glszm SmallAreaLowGrayLevelEmphasis, squareroot glcm Imc1, wavelet LHH firstorder Entropy, wavelet LHH ngtdm Busyness, wavelet LLH firstorder Entropy, wavelet LLH firstorder InterquartileRangewavelet LLL glcm Idm and wavelet LLL glszm SizeZoneNonUniformityNormalized (**Figure 5**).

**Table 1:** The number of features extracted.

Feature type	Number
first-order	306
glcm	374
gldm	238
glrlm	272
glszm	272
ngtdm	85
shape	14

The number of features extracted by LASSO model.

### 3.2 RF model was the optimal classifier

**Figure 6:**
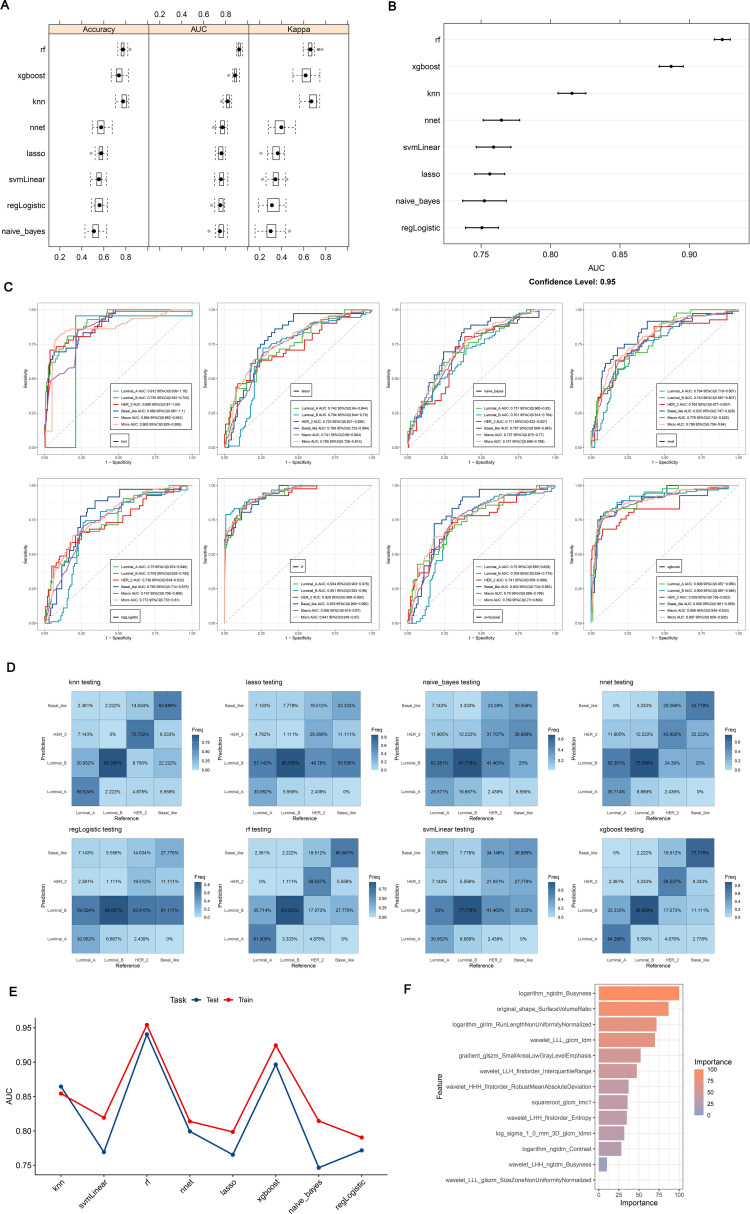
**(A, B)** ROC curves and confusion matrices for 8 machine algorithms; **(C)** The test set verifies the ROC curve for each model separately; **(D)** The test set verifies each model confusion matrix separately; **(E)** Test set and verification set accuracy line chart curve; **(F)** Feature importance ranking of Random Forest. **(A, B)** Random Forest has the highest ROC Area under Curve (AUC) value; **(C)** ROC is a one-to-many (OvR) multi-class strategy, also known as one-to-many, which involves calculating each class. At each step, the given class is treated as a positive class, and the remaining classes are treated as a negative class of the whole. ‘macro’: calculates the metrics for each label and finds their unweighted average. This does not take into account label imbalance. ‘micro’: calculates the global indicator by counting total true positives, false negatives, and false positives; **(D)** Confusion matrix is a situation analysis table that summarizes the prediction results of classification model in machine learning, and summarizes the records in the data set in the form of matrix according to the real category and the category judgment predicted by the classification model; **(E)** AUC for different test sets and validation sets; **(F)** The importance of features of Random Forest model corresponds to the scores displayed by corresponding color cards.

In sum of 8 ML models were constructed in the training set to select the best classifier to accurately predict the BC subtypes. Among 8 ML models, RF possessed the highest AUC value and accuracy in training set (**Figures 6A, B**), As presented in training and testing sets, the RF model retained the highest AUC values for the four BC subtypes, as did the Macro average AUC and Micro average AUC values, demonstrating the excellent predictive capacity of RF (**Figure 6C**). In addition, the abilities of 8 ML models were assessed using confusion matrix, highlighting RF had the highest accuracy, sensitivity, recall and F1 score in 8 ML models (**Table 2**, **Figure 6D**). The line graph illustrating the AUC discrepancies of 8 ML models between training and testing sets emphasized the highest AUCs of RF in both sets (**Figure 6E**). In conclusion, RF was the optimal classifier predicting image-omics LASSO features of BC. As a consequence,14 LASSO features were sorted by Random Forest model according to their importance. Among these, logarithm ngtdm Busyness and original shape Surface Volume Ratio contributed the most to the model due to their higher importance values (**Figure 6F**).

**Table 2:** Model energy efficiency index.

Model	Class	Sensitivity	Specificity	Pos Pred Value	Neg Pred Value	Precision	Recall	F1
knn	Class: Luminal_A	0.595	0.964	0.806	0.904	0.806	0.595	0.685
knn	Class: Luminal_B	0.956	0.790	0.775	0.959	0.775	0.956	0.856
knn	Class: HER_2	0.707	0.964	0.829	0.931	0.829	0.707	0.763
knn	Class: Basal_like	0.639	0.948	0.719	0.927	0.719	0.639	0.676
svmLinear	Class: Luminal_A	0.310	0.946	0.591	0.845	0.591	0.310	0.406
svmLinear	Class: Luminal_B	0.778	0.580	0.583	0.775	0.583	0.778	0.667
svmLinear	Class: HER_2	0.220	0.893	0.333	0.824	0.333	0.220	0.265
svmLinear	Class: Basal_like	0.389	0.850	0.350	0.870	0.350	0.389	0.368
rf	Class: Luminal_A	0.619	0.970	0.839	0.910	0.839	0.619	0.712
rf	Class: Luminal_B	0.933	0.731	0.724	0.935	0.724	0.933	0.816
rf	Class: HER_2	0.585	0.982	0.889	0.907	0.889	0.585	0.706
rf	Class: Basal_like	0.667	0.936	0.686	0.931	0.686	0.667	0.676
nnet	Class: Luminal_A	0.357	0.946	0.625	0.854	0.625	0.357	0.455
nnet	Class: Luminal_B	0.756	0.655	0.624	0.780	0.624	0.756	0.683
nnet	Class: HER_2	0.439	0.857	0.429	0.862	0.429	0.439	0.434
nnet	Class: Basal_like	0.528	0.913	0.559	0.903	0.559	0.528	0.543
lasso	Class: Luminal_A	0.310	0.964	0.684	0.847	0.684	0.310	0.426
lasso	Class: Luminal_B	0.856	0.462	0.546	0.809	0.546	0.856	0.667
lasso	Class: HER_2	0.293	0.958	0.632	0.847	0.632	0.293	0.400
lasso	Class: Basal_like	0.333	0.896	0.400	0.866	0.400	0.333	0.364
xgboost	Class: Luminal_A	0.643	0.952	0.771	0.914	0.771	0.643	0.701
xgboost	Class: Luminal_B	0.889	0.790	0.762	0.904	0.762	0.889	0.821
xgboost	Class: HER_2	0.585	0.958	0.774	0.904	0.774	0.585	0.667
xgboost	Class: Basal_like	0.778	0.942	0.737	0.953	0.737	0.778	0.757
naive_bayes	Class: Luminal_A	0.286	0.892	0.400	0.832	0.400	0.286	0.333
naive_bayes	Class: Luminal_B	0.678	0.597	0.560	0.710	0.560	0.678	0.613
naive_bayes	Class: HER_2	0.317	0.821	0.302	0.831	0.302	0.317	0.310
naive_bayes	Class: Basal_like	0.306	0.908	0.407	0.863	0.407	0.306	0.349
regLogistic	Class: Luminal_A	0.310	0.958	0.650	0.847	0.650	0.310	0.419
regLogistic	Class: Luminal_B	0.867	0.387	0.517	0.793	0.517	0.867	0.647
regLogistic	Class: HER_2	0.195	0.964	0.571	0.831	0.571	0.195	0.291
regLogistic	Class: Basal_like	0.278	0.919	0.417	0.859	0.417	0.278	0.333

The test set accuracy, sensitivity, recall and F1 of Random Forest(RF) are greater than those of other models.

### 3.3 Using the nomogram model to predict BC

**Figure 7:**
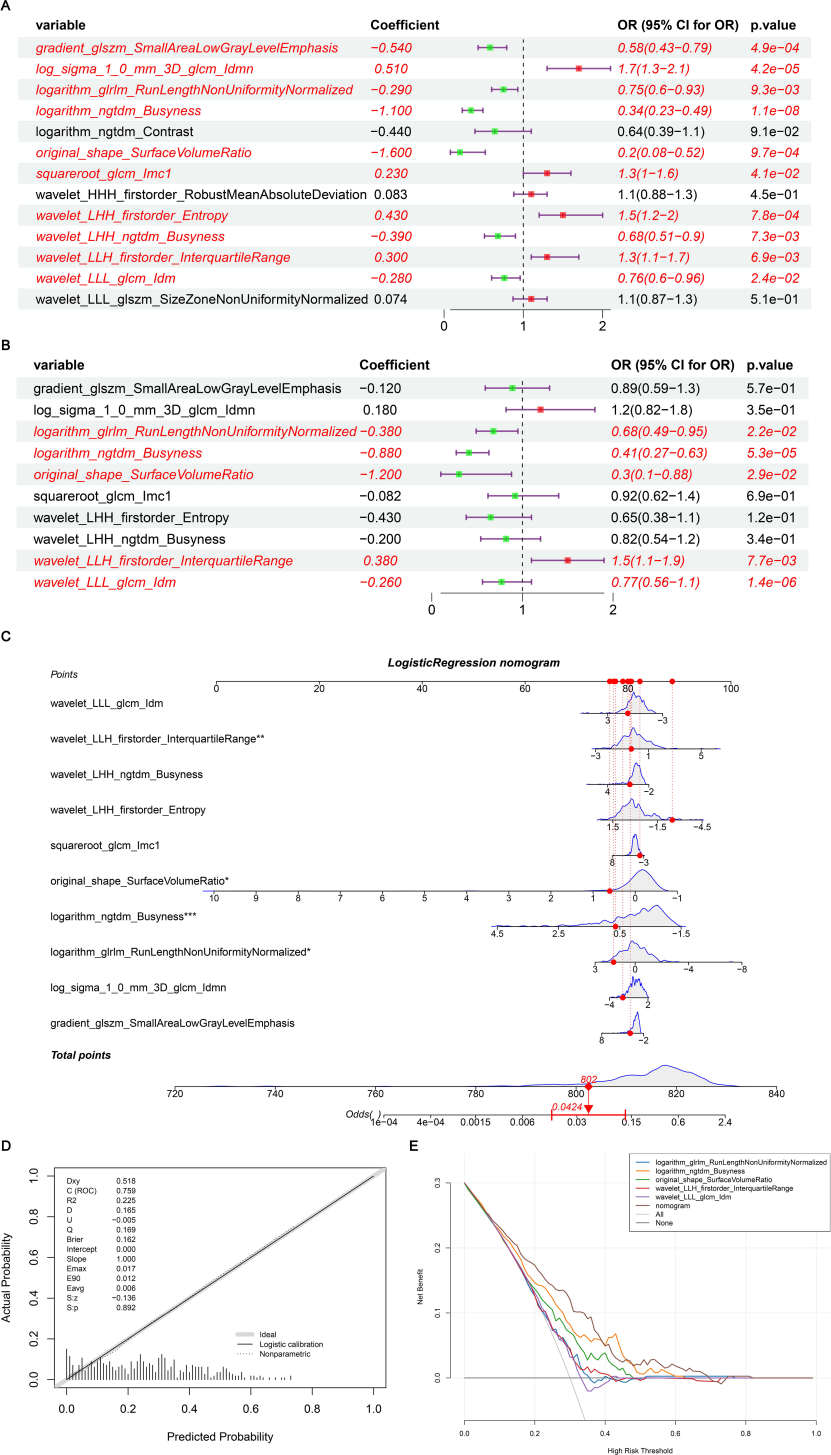
**(A)** Single factor logistic regression model forest map; **(B)** Multifactor logistic regression model forest map; **(C)** The nomogram predicted the relative risk of patients; **(D)** Nomogram calibration curve; **(E)** Decision curve. **(A)** A single factor logistic regression model was constructed based on the 14 image omics features of the training set, and the risk forest map was drawn according to the model. The results of 10 features in the model were significant (*p* < 0.05); **(B)** A multi-factor logistic regression model was constructed based on the 14 image-omics features of the training set, and the risk forest map was drawn according to the model. The results of 5 features in the model were significant (*p* < 0.05); **(C)** Multivariate logistic regression analysis was performed to obtain five significant factors and a column graph was constructed. Each factor corresponds to a score. The total score of each factor was added to correspond to the total score, and the relative risk (Odds Ratio) of the patient was predicted according to the total score; **(D)** Based on the above nomogram prediction model, the calibration curve was drawn. The closer the slope is to 1, the more accurate the prediction is. In addition, the c-index of the model was 0.759, and the corrected c-index was 0.732, indicating that the column-line model was well fitted and the prediction results of our logistic regression model were quite good, which can be used in clinical diagnosis; **(E)** The horizontal coordinate is the threshold probability: In the risk assessment tool, the probability that patient i is diagnosed with the disease is denoting Pi; When Pi reaches a certain threshold (denoted as Pt), the case is defined as positive and treatment is administered. There will be patient benefit (benefit), non-patient harm (harm) and patient loss (harm) if the patient is not treated. The ordinate is the Net Benefit (NB) after subtracting the disadvantages from the advantages. In addition to the curved lines that represent different models of clinical diagnosis (identified by the legend), there are two lines that represent the two extremes. The horizontal one indicates that all samples are negative (Pi < Pt), all are untreated, and the net benefit is 0. The slanted one means that all the samples were positive, all of them were treated, and the net benefit is a negative backslash. As can be seen from the figure, within the Pt [0-1] interval, the benefits of the imaging features, clinical features and Nomogram are all higher than those of the extreme curves, so the optional Pt ranges are relatively large and safe.

To identify BC features for nomogram modeling, univariate Cox regression analysis was performed, followed by multivariate Cox analysis. Ten LASSO-selected features (*p* < 0.05) from the univariate analysis were subsequently incorporated into the multivariate model (**Table 3**, **Figure 7A**), yielding 5 crucial features, namely logarithm glrlm RunLengthNonUniformityNormalized, logarithm ngtdm Busyness, original sape SurfaceVolumeRatio, wavelet LLH firstorder InterquartileRange and wavelet LLL glcm Idm (**Table 4**, **Figure 7B**). A nomogram model embracing 5 crucial features was developed immediately; the OR values of BC patients increased with the elevated total points (**Figure 7C**). Importantly, the validity and universality of the nomogram model were certified via calibration curve and DCA curve. The calibration curve manifested that the slope of the nomogram model almost achieved to 1. In addition, the c-index of 0.732 after model correction was close to the c-index of 0.759 (**Figure 7D**). Further DCA demonstrated that the nomogram model outweighed any single crucial feature by providing a superior net benefit (**Figure 7E**).

**Table 3:** Single factor logistic regression model.

Variable	Coefficient	OR (95% CI for OR)	p.value
gradient_glszm_SmallAreaLowGrayLevelEmphasis	-0.54	0.58(0.43-0.79)	0.00049
log_sigma_1_0_mm_3D_glcm_Idmn	0.51	1.7(1.3-2.1)	4.20E-05
logarithm_glrlm_RunLengthNonUniformityNormalized	-0.29	0.75(0.6-0.93)	0.0093
logarithm_ngtdm_Busyness	-1.1	0.34(0.23-0.49)	1.10E-08
logarithm_ngtdm_Contrast	-0.44	0.64(0.39-1.1)	0.091
original_shape_SurfaceVolumeRatio	-1.6	0.2(0.08-0.52)	0.00097
squareroot_glcm_Imc1	0.23	1.3(1-1.6)	0.041
wavelet_HHH_firstorder_RobustMeanAbsoluteDeviation	0.083	1.1(0.88-1.3)	0.45
wavelet_LHH_firstorder_Entropy	0.43	1.5(1.2-2)	0.00078
wavelet_LHH_ngtdm_Busyness	-0.39	0.68(0.51-0.9)	0.0073
wavelet_LLH_firstorder_InterquartileRange	0.3	1.3(1.1-1.7)	0.0069
wavelet_LLL_glcm_Idm	-0.28	0.76(0.6-0.96)	0.024
wavelet_LLL_glszm_SizeZoneNonUniformityNormalized	0.074	1.1(0.87-1.3)	0.51

The odd ratio (OR) value is the relative risk, also known as the odds ratio, which refers to the ratio of the exposed and non-exposed people in the case group divided by the ratio of the exposed and non-exposed people in the control group.

**Table 4:** Multiple logistic regression models.

Variable	Coefficient	OR (95% CI for OR)	p.value
gradient_glszm_SmallAreaLowGrayLevelEmphasis	-0.12	0.89(0.59-1.3)	0.57
log_sigma_1_0_mm_3D_glcm_Idmn	0.18	1.2(0.82-1.8)	0.35
logarithm_glrlm_RunLengthNonUniformityNormalized	-0.38	0.68(0.49-0.95)	0.022
logarithm_ngtdm_Busyness	-0.88	0.41(0.27-0.63)	5.30E-05
original_shape_SurfaceVolumeRatio	-1.2	0.3(0.1-0.88)	0.029
squareroot_glcm_Imc1	-0.082	0.92(0.62-1.4)	0.69
wavelet_LHH_firstorder_Entropy	-0.43	0.65(0.38-1.1)	0.12
wavelet_LHH_ngtdm_Busyness	-0.2	0.82(0.54-1.2)	0.34
wavelet_LLH_firstorder_InterquartileRange	0.38	1.5(1.1-1.9)	0.0077
wavelet_LLL_glcm_Idm	-0.26	0.77(0.56-1.1)	1.40E-06

The odd ratio(OR) value is the relative risk, also known as the odds ratio, which refers to the ratio of the exposed and non-exposed people in the case group divided by the ratio of the exposed and non-exposed people in the control group.

## 4 Discussion

BC has the highest incidence rate among all female cancers globally. The use of imaging genomics and machine learning to construct novel cancer diagnostic models has been widely applied, but there has been no complete report on its application in BC. In our study, we developed a model with improved predictive performance based on the specific molecular subtypes of BC to meet the individualized treatment needs. We confirmed that radiomics characteristics derived from DBT can predict the manifestations of different molecular types of BC, thus providing more value and information for patient personalized treatment. In this study, we integrated imagomics with machine learning to uncover five novel key features of BC. Based on these findings, we constructed a nomogram model capable of predicting the risk level in BC patients with acceptable accuracy. Furthermore, we devised a combined radiomic model that integrates the radiomic features derived from DBT with IHC results for personalized risk prediction. This approach fully underscores the necessity and clinical significance of establishing a robust BC risk prediction model. Compared with clinical radiological nomogram, combined radiomic nomogram has superior prognostic performance in patients with different molecular types of BC.

Recent studies have revealed that the application of radiomics holds promising potential in enhancing tumor prognosis. Notably, research has demonstrated that radiomics-based nomograms can effectively predict the efficacy of neoadjuvant chemotherapy in BC patients, utilizing pre-treatment magnetic resonance imaging as a foundation (30, 31). In addition, radiomics signature (Rad-score) was used to predict DFS in HER-2 positive invasive BC receiving neoadjuvant chemotherapy, which may be used to personalize treatment strategies (32). Exploration of tumor heterogeneity by radiomics can be an alternative to genomic and transcriptomic analysis (16, 33, 34). Radiomics of magnetic resonance imaging has shown high performance and remains valid for radiomics of mammography—a finding of great importance for studies related to DBT (17, 35). Studies by Ma et al. (17) and Zhang et al. (35) have demonstrated high accuracy in differentiating TN BC subtypes, with Ma’s approach showing optimal TN discrimination (alongside HER2 and luminal subtypes), while Zhang’s radiomics-based method achieved comparable performance in digital mammography. However, all analyses relied on DM imaging, and the replicability of these findings using DBT remains uncertain. Some studies (7) have proposed using synthetic mammography instead of original DBT images to plot ROI on synthetic mammography in clinical practice, and suggested that it is impractical to plot ROI on original DBT images, and the reproducibility of ROI on original DBT images will be limited. Although synthetic mammography may lose some tomographic data, based on the current research status of DBT, a radiomic model was constructed in this study. A total of 1175 imaging features (candidate features) were extracted based on the fusion of 433 DBT images in 4 groups of BC subtypes (luminal A-subtype, luminal B-subtype, HER2-positive and basal-like BC). We identified five novel key features of BC by integrating imagomics and machine learning. Our study presents several significant advantages over previous studies by constructing an ensemble learning model based on mammography and IHC through radiomic analysis of mammography to predict risk models based on molecular subtypes, thus providing enhanced value for personalized treatment. In contrast, prior studies solely relied on routine clinical and radiological features, lacked precise subtype analyses, or utilized only imaging omics methods.

In the realm of medical imaging holography, machine-learning methods hold the potential to attain greater precision while integrating diverse types of information for a broad array of applications, such as disease diagnosis and prognosis evaluation. Research (8) has indicated that machine-learning models possess a marginally superior edge over traditional risk factor-based models in predicting future BC risk. Furthermore, neural network-based BC risk prediction models that incorporate imaging features demonstrate outstanding performance. This finding implies that the integration of imaging inputs within machine-learning models can provide more precise breast cancer risk prediction. Prior BC risk assessments have already acknowledged the significance of imaging features in mammography (9, 36). Nevertheless, the existing model was grounded on the underlying pattern visually assessed by radiologists, and the whole image was subjectively summarized as a density score on mammography as the model input (37). Some studies have developed a novel LASSO-logic modeling approach to perform initial variable screening and eliminate relatively insignificant coefficients of independent variables in the model (38). Thus, regression analysis effectively addresses variable collinearity, particularly in high-dimensional screening scenarios (39). 8 machine learning models were referenced in this study, with the LASSO model used to identify features strongly correlated with BC (LASSO features). LASSO logistic regression was then applied to each mammary gland category, and the category with the highest probability was selected through 10x cross-validation calculations for classification. Following a comprehensive parameter analysis, the random forest algorithm was chosen as the best performing machine learning method. The optimal algorithm was determined and the image holographic score (Rad_score) was calculated. The features of the random forest model were ranked by importance, resulting in 14 significant image group features.

In oncology research, nomogram models utilizing multivariate regression analysis (particularly logistic/Cox regression) are widely adopted for predicting clinical outcomes such as tumor recurrence, metastasis, and mortality (40–42). These tools transform identified risk factors into visual scoring systems, with multivariate regression serving as their computational foundation. Compared to conventional methods, nomograms provide enhanced predictive accuracy and interpretability (42, 43), as evidenced by their capacity to quantify variable contributions through graphical outputs. Our implementation aligns with established methodological frameworks in the field: we constructed predictive models and nomographs based on patients’ risk factors and verified their accuracy and validity to predict the risk of these patients, and evaluated the diagnostic accuracy and clinical value of the models using decision curve analysis. Although this model has certain predictive capabilities, it is not yet suitable for standalone clinical decision-making, such as replacing invasive biopsy to confirm subtypes. Instead, its primary clinical value lies in providing complementary information to guide preliminary treatment planning until the accuracy is further improved.

This study has several limitations that warrant discussion. First, the inherent constraints associated with its single-center retrospective design must be acknowledged, which may limit the generalizability of our findings to other populations or institutions due to potential variations in patient demographics, imaging protocols, and pathological practices. Second, the training and validation of the five key features necessitated a vast amount of medical image data. During this process, machine learning algorithms might absorb biases present in the data, potentially leading to skewed prediction outcomes. Nevertheless, our commitment to the subtype research and diagnosis of BC remains unwavering. Third, we did not analyze the morphological characteristics of the four subtypes in this study, leaving room for future studies to explore this relevant content. And also, we did not handle the potential class imbalance among the four BC subtypes in the model training. Strategies should be implemented to further improve the model’s robustness and generalizability across all subtypes. Fourth, radiomics features were extracted based on manually-drawn ROIs. To mitigate potential issues, features with poor inter-observer reproducibility were excluded from the analysis. Fifth, another limitation of this study is the lack of external validation on an independent cohort, which would strengthen the generalizability of our findings; future studies should include multi-center external validation to confirm model robustness. Sixth, although the radiomic features identified exhibit statistical significance for predicting BC subtypes, their specific pathophysiological implications remain unclear, resulting in limited clinical interpretability. Seventh, although this study constructed a risk model applicable to the clinical diagnosis of BC patients and screened five key features for constructing the nomogram through univariate and multivariate regression analysis, which does possess certain clinical value, we will continue to expand the sample size in subsequent studies to further verify these key features.

## 5 Conclusions

In summary, this study analyzed Luminal A, Luminal B, HER-2 positive and TN types of BC patients by means of imaging omics analysis and a variety of machine learning methods, Based on our validation results, these models demonstrate high reproducibility in BC patients. Additionally, we have identified potential prognostic variables in patients with BC, with the aim of identifying an optimal classification model and providing new insights for the diagnosis and clinical treatment of BC.

## Data Availability

The original contributions presented in the study are included in the article/supplementary material. Further inquiries can be directed to the corresponding authors.
